# Link between Plant Phosphate and Drought Stress Responses

**DOI:** 10.34133/research.0405

**Published:** 2024-07-01

**Authors:** Nidhi Kandhol, Sangeeta Pandey, Vijay Pratap Singh, Luis Herrera-Estrella, Lam-Son Phan Tran, Durgesh Kumar Tripathi

**Affiliations:** ^1^ Crop Nanobiology and Molecular Stress Physiology Lab, Amity Institute of Organic Agriculture, Amity University Uttar Pradesh, Sector-125, Noida 201313, India.; ^2^ Plant Microbe Interaction Lab, Amity Institute of Organic Agriculture, Amity University Uttar Pradesh, Sector-125, Noida 201313, India.; ^3^ Plant Physiology Laboratory, Department of Botany, C.M.P. Degree College, A Constituent Post Graduate College of University of Allahabad, Prayagraj 211002, India.; ^4^ Unidad de Genomica Avanzada, Centro de Investigación y de Estudios Avanzados del Intituto Politecnico Nacional, Irapuato 36821, Mexico.; ^5^ Institute of Genomics for Crop Abiotic Stress Tolerance, Department of Plant and Soil Science, Texas Tech University, Lubbock TX 79409, USA.

## Abstract

The menace of drought has persistently loomed over global crop production, posing a serious threat to agricultural sustainability. Research on drought stress highlights the important role of the phytohormone abscisic acid (ABA) in orchestrating plant responses to drought conditions. ABA regulates various drought/dehydration-responsive genes, initiates stomatal closure, and influences cellular responses to drought stress. Additionally, plants employ a phosphate starvation response (PSR) mechanism to manage phosphate (Pi) deficiency, with ABA playing a role in its regulation. However, despite intensive research in these fields, the precise connection among PSRs, drought stress, and ABA signaling still needs to be determined. Recently, PSR-related gene induction has been reported to occur before the induction of ABA-responsive genes under progressive mild drought. Mild drought decreases Pi uptake and contents in plants, triggering PSRs, which play an important role in plant growth during mild drought. Both ABA-responsive and PSR-related gene expression could indicate plant perception of external moisture conditions. Thus, integrating the information regarding their associated gene expression with soil moisture contents and thermographic data can enable timely irrigation optimization to mitigate the effect of drought on crop productivity.

## Introduction

Throughout the annals of agricultural history, global crop production has been imperiled by drought. This natural disaster is intricate and capricious, exhibiting a wide range of diverse characteristics concerning intensity, timing, duration, and occurrence patterns [1]. Many research studies, predominantly conducted on the model experimental plant *Arabidopsis thaliana*, elucidated the pivotal role of the phytohormone abscisic acid (ABA) in plant responses to abiotic stresses [2,3]. ABA is a crucial regulator in orchestrating plant responses toward a broad spectrum of drought conditions and in adjusting plant water utilization [4]. In plants, drought stress triggers an increase in endogenous ABA levels. Elevated ABA levels regulate numerous dehydration-responsive genes and initiate signal transduction pathways, leading to stomatal closure and various cellular responses to drought conditions [5]. Furthermore, plants evolved a phosphate (Pi) starvation response (PSR) mechanism designed to detect Pi deficiency and subsequently adjust root and stem growth [6,7]. ABA, along with other phytohormones, is involved in the transcriptional regulation of genes associated with the PSRs in plants [8]. Moreover, ABA- and/or drought stress-responsive *cis*-regulatory elements like ABA-responsive element, dehydration-responsive element, and MYB-binding site motif are commonly found in the promoters of most Pi transporter-encoding genes [8]. Understanding the PSRs within the plant system in response to drought stress is crucial, as Pi limitations frequently constrain plant growth. Plants reduce Pi uptake as soil moisture declines [9]. Interestingly, the exogenous application of Pi can enhance plant drought tolerance through physio-biochemical adjustments [10]. Despite existing reports indicating a connection between the PSRs and drought responses, the exact link remained relatively unclear until recently. In their current research, Nagatoshi et al. [11] employed field-to-lab-oriented research to unravel that the mechanism governing plant responses to mild-drought stress in the field is related to the induction of PSRs (Figure).

**Figure.**
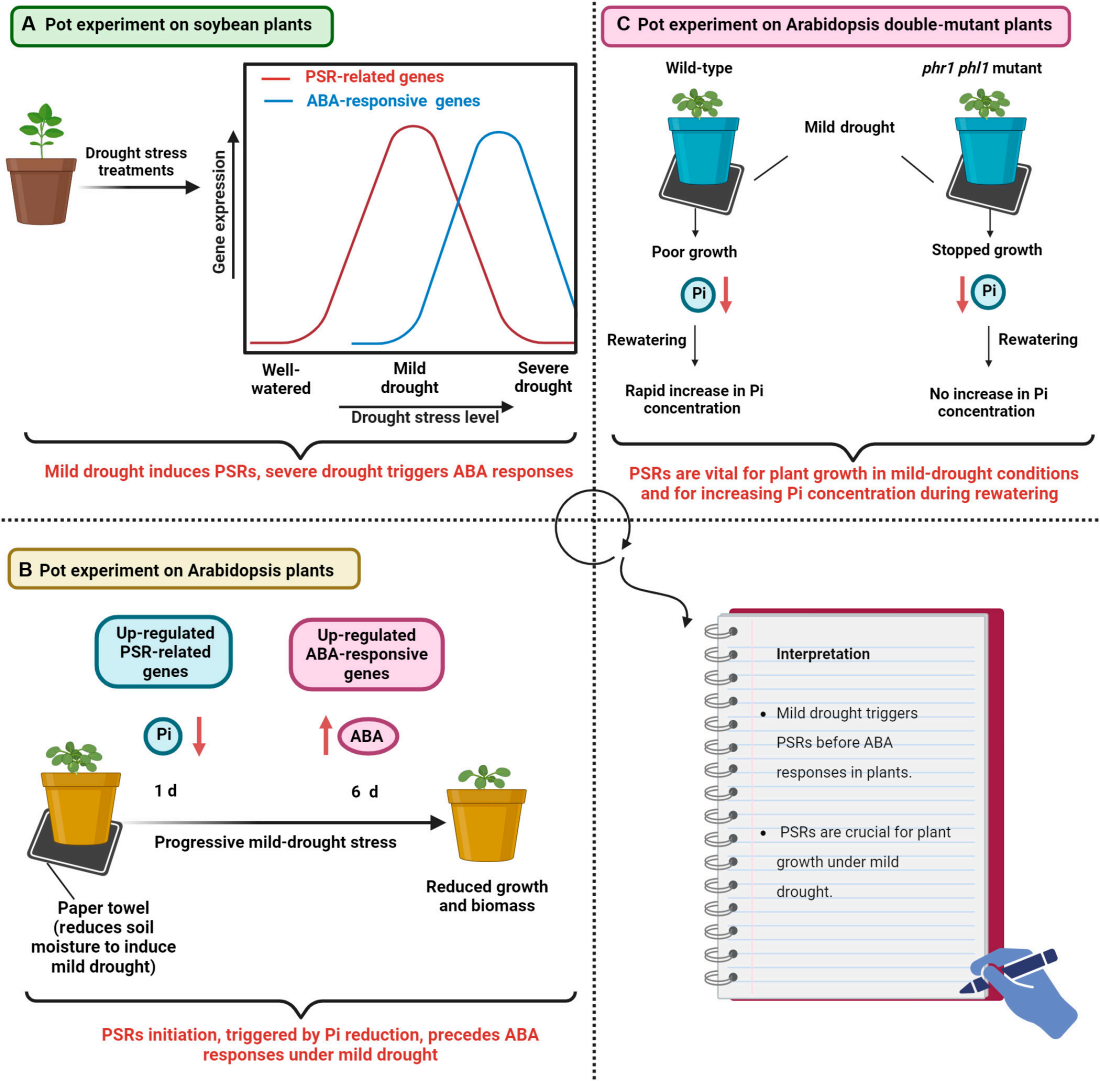
Sequential activation of the phosphate (Pi) starvation responses (PSRs) and abscisic acid (ABA) responses in soybean (*Glycine max*) and Arabidopsis (*Arabidopsis thaliana*) plants under mild-drought conditions. (A) In potted soybean plants, as drought intensity increases, the up-regulation of PSR-related gene expression occurs first, followed by the escalation of ABA-responsive gene expression under severe-drought conditions. (B) In potted Arabidopsis plants, the reduction in soil moisture to induce mild drought leads to a similar pattern: up-regulation of PSR-related gene expression precedes the up-regulation of ABA-responsive gene expression, confirming the findings observed in soybean. (C) Under mild drought, the Arabidopsis double mutant *phr1 phl1*, lacking the PSR mechanism, stopped to grow, in contrast to the continuous growth, even poorly, observed in wild-type plants. This phenomenon highlights the vital role of PSRs in regulating plant growth under mild-drought stress. Upon rewatering, wild-type plants quickly elevated Pi concentrations, while the *phr1 phl1* double mutant displayed no significant increase, indicating the essential role of PSRs in the rapid rise of Pi concentration in plants during rehydration.

## Dynamics of PSRs and ABA Signaling in Soybean (*Glycine max*) under Mild-Drought Stress

Nagatoshi et al. [11] used ridges as a valuable experimental method for simulating mild-drought stress in field-grown soybean plants. The RNA-sequencing analysis of soybean leaves subjected to mild-drought stress revealed significant differences in gene expression, with 11.4% of the up-regulated genes identified as PSR genes. This finding indicated that the expression of PSR marker genes was stimulated in soybean plants under mild-drought conditions. Notably, PSR-related genes were up-regulated earlier than ABA-responsive genes, suggesting their potential as early indicators of mild-drought stress. Mild drought reduced the concentrations of Pi in soybean leaves, with PSR-related gene expression being inversely correlated with Pi levels. This reduction in Pi levels under mild-drought conditions was consistent with the observations from field and pot trials. In addition to Pi, other inorganic nutrients, including nitrogen (N) and micronutrients, were also decreased by mild drought; however, the reduction in Pi levels was more pronounced. Pot trials, incorporating 5 distinct volumetric water content levels to induce varying degrees of water deficit, confirmed the link between soil water status and PSR activation, with increasing drought severity decreasing PSR-related gene expression. Furthermore, the transition from PSR activation to ABA responses was observed as drought severity increased beyond a certain threshold, indicating a sequential response mechanism to drought. Under severe-drought conditions, the induction of PSRs was inhibited due to a relative increase in Pi concentration resulting from reduced leaf water content. Moreover, ABA content and the expression of ABA-responsive genes increased under severe-drought stress. ABA initiates a response when water loss from the leaves occurs, accompanied by decreased osmotic potential [12]. Conclusively, in soybean plants, mild drought caused the PSRs followed by ABA responses, and ABA responses are solely induced by severe drought (Figure).

## Sequential Activation of PSRs and ABA Signaling in Arabidopsis under Progressive Mild-Drought Stress

The authors also confirmed these observations using Arabidopsis employing a progressive mild-drought induction method over 6 d, wherein control plants were kept in pots submerged in water while those exposed to drought stress were placed on paper towels to reduce soil moisture gradually. RNA sequencing of Arabidopsis shoots under mild-drought stress also identified differential expression of many genes, with up to 33% of the up-regulated genes being PSR-related. Hierarchical clustering revealed distinct gene clusters associated with PSRs and ABA responses. PSR-related genes were predominantly up-regulated after 1 d of drought treatment, whereas ABA-responsive genes were activated later, after 6 d of drought treatment. This sequential activation pattern indicates that PSR induction precedes ABA responses under mild-drought conditions. Reduced Pi concentrations were observed in drought-treated Arabidopsis plants, indicating decreased Pi uptake under mild-drought conditions. Rehydration experiments rapidly restored Pi concentration and decreased PSR-related gene expression, suggesting that mild-drought-induced reduction in Pi levels was associated with soil moisture conditions rather than inhibition of plant growth caused by mild drought. These findings concur with the notion that mild-drought stress inhibits Pi uptake, leading to reduced Pi concentrations and subsequent PSR induction in plants. Inducing mild-drought stress in Arabidopsis *phr1 phl1* double-mutant plants [13], deficient in PSRs, revealed lower Pi concentrations in both mutant and wild-type plants compared with the control treatment. However, wild-type plants, but not the *phr1 phl1* double mutant, showed a quick rise in Pi contents upon rewatering, highlighting the role of PSRs in restoring Pi levels. Furthermore, the PSR-deficient mutant ceased growth under mild drought, contrasting with the continued, albeit limited, growth observed in wild-type plants, emphasizing the crucial role of PSRs in sustaining plant growth under progressive mild drought (Figure).

## Conclusion and Future Perspectives

The findings of Nagatoshi et al. [11] from soybean and Arabidopsis experiments reveal that mild drought hampers Pi uptake and reduces leaf Pi concentration, ultimately inducing PSRs in plants. Soil-Pi deficiency also triggers PSR activation in plants, stimulating root alterations that expand the root–soil interface to enhance Pi uptake, while simultaneously regulating above-ground growth to ensure optimal seed viability [14]. Therefore, further research is essential to uncover the molecular mechanisms that govern PSR-induced growth adjustments in plants under mild drought, as well as to discern any differences between the mechanisms underlying soil-Pi deficiency-induced PSRs and those regulating mild-drought-induced PSRs. The discussed study underscores the potential of PSR-related genes as early indicators of mild-drought stress responses in plants. Investigating how external water conditions affect intracellular Pi dynamics and PSR-related gene expression could provide valuable insights into plant responses to combined stresses. Future studies should focus on elucidating the timing and regulatory networks associated with early activation of PSR-related genes, potentially identifying novel drought and Pi deficiency biomarkers. PSRs and ABA responses serve as distinct stress-responsive mechanisms in plants. The sequence of PSRs and ABA responses under drought varies, and a clear correlation between them is still awaiting confirmation. Moreover, the presence of this pattern in woody and other advanced plants needs validation. Furthermore, rehydration experiments postdrought exposure could offer comprehensive insights into plant recovery mechanisms. Understanding the molecular pathways involved in postdrought recovery can inform strategies to enhance crop resilience and productivity. Moreover, simultaneous Pi deficiency and drought stress amplify their individual adverse effects on plant growth, as documented by Meier et al. [15,16] in wheat (*Triticum aestivum*) genotypes evaluated under varied irrigation and Pi levels. Different wheat genotypes exhibited significant variability in water and Pi utilization under combined stress likely due to their differences in agronomic traits. By uncovering the intricate molecular interactions between PSRs and plant responses to the deficiencies of other nutrients like nitrogen, sulfur, zinc, and iron, as well as the relationship between plant responses to nutrient deficiencies and to drought, we can pave the way for developing resilient agricultural systems capable of withstanding environmental challenges, including drought and/or nutrient deficiencies. Additionally, exogenous Pi application, such as through the widely used phosphoric acid [17], also induces vital physiological adjustments for drought tolerance like enhanced root conductivity and altered root architecture [18,19]. However, excessive phosphoric acid usage may exacerbate crop sensitivity to drought by disturbing soil nutrient balance and worsening soil acidification. While exogenous Pi donors may offer advantages for drought tolerance, careful management is pivotal to prevent their adverse effects on soil and plant health. Furthermore, the connection between exogenous nutrient supplementation and ABA responses needs more investigations, requiring molecular assays to explore their interaction and potential effects of nutrient supplementation on ABA gene expression. Interdisciplinary research is crucial for advancing our understanding of molecular reactions to mild drought and the interplay between plant responses to nutrient deficiencies and to drought.
